# Observations on the Illusions of the Insane, and on the Medico-Legal Question of Their Confinement

**Published:** 1833-10-01

**Authors:** 


OBSERVATIONS ON THE ILLUSIONS OF THE INSANE, AND ON THE MEDICO-LEGAL QUESTION OF THEIR CONFINEMENT. Translated from the French of *M. Esquirol*, Medecin en Chef de la Maison Royale de Charenton, Membre du Conseil de Salubrité, &c. &c. &c. by *William Liddell*, Member of the Royal College of Surgeons, &c. Octavo, pp. 89. Renshaw and Rush, 1833.

ESQUIROL has long been at the head of the mad-doctors of Europe, and as he had more ample means of gaining information than almost any other individual now living, his observations must always be received with great respect and attention on the subject of insanity. M. Esquirol objects to the French laws respecting lunatics, and his translator is not satisfied with those which have been enacted on this side of the Channel. Esquirol complains that the existing law has in view rather the maintenance of public order, and the preservation of the fortune of the insane, than their restoration to liberty. Mr. Liddell criticises the English law which makes great ceremony, and throws great trouble in the way of a madman's reception into a public asylum, but requires no document or return for twelve months after a person has been immured in private confinement—perhaps without being insane at all.

But leaving these medico-legal questions to the collective wisdom of our senates, we come to purely medical subjects. We shall open our analysis with the following passage.

" Insane persons fancy they see, hear, smell, taste, and touch, although external objects are not presented to their senses, and are, consequently, incapable of producing any impression upon them. This symptom is an intellectual phenomenon, totally independent of the organs of sense, and takes place although they may be inactive, or have even ceased to exist. Thus, there are deaf persons who fancy they hear, blind ones who think they see, &c. &c. The ancients had only observed this symptom, as far as it related to the remembrance of the sensations of sight, and had given it the name of *Vision*. But the analysis of the thoughts of the insane, for they do think and reason, has proved to me that this phenomenon is produced by the action of the brain, reacting upon the sensations previously received by the other senses, as well as by that of sight. This has led me to give to this phenomenon the generic name of *Hallucinations*. In the same paper in which I pointed out one of the most remarkable psychological phenomena of delirium, I related some facts which shew that the hallucinations alone, sometimes, characterize a variety of monomania." 2.

M. Esquirol makes a marked distinction between hallucinations, or visions, and illusions.

" In hallucinations, every thing passes within the brain: visionaries, and persons under the influence of extatic impressions, are hallucinarians; they dream even when they are awake. The activity of the brain is so energetic in them, that they give form and reality to the images which the memory re-produces, without the aid of the senses.

In illusions, on the contrary, the sensibility of the nervous extremities is excited, the senses are active, and actual impressions produce the reaction of the brain. This reaction being under the influence of the ideas and passions, which govern the insane, they are deceived as to the nature and cause of their actual sensations. Illusions are not uncommon in a state of health, but reason dissipates them. A square tower seen from a distance appears round, but if we approach it, the error is soon rectified. When we travel amongst mountains we often take them for clouds, but on looking attentively, the error is dissipated. To him, who is in a boat, the bank appears to move, reflection immediately destroys the illusion." 3.

Hypochondriacs, he observes, have illusions which arise from the internal senses. They deceive themselves with respect to the intensity of their feelings; but do not attribute their ailments to absurd causes, nor talk irrationally, unless affected with melancholia in addition, when there is delirium.

" Two conditions are necessary for the perception of a sensation; the soundness of the organ which receives the impression, and the soundness of the instrument that reacts upon it.

The illusions of the senses recognize, also, two causes; a disordered state of the senses, and a disordered state of the brain.

If the sensibility and activity of the organs are disturbed, it is evident that the impressions made upon the senses, by external objects, are modified; and if, at the same time, the brain is in a state of disease, it is incapable of rectifying the errors of the senses. From these causes arise illusions." 4.

The passions, which produce so many illusions among the sane, modify also the impressions of the insane, and are the cause of a thousand illusions.

From these preliminary observations, M. Esquirol proceeds to practical illustrations, several of which we shall condense.

1. The famous Térouane de Méricourt, lived ten years in the Salpêtrière in a state of insanity. She used to throw two pails of water on her bed every morning and evening, and lie down in it immediately afterwards. Some of the insane have such excessive sensibility of the skin, that the slightest touch appears to them like a deadly blow.

2. An officer was seized with an intermittent in the Prussian campaign. They gave him a glass of brandy with gun-powder in it. He became immediately insane. He lay down upon straw, but fancied the straws were the beaks of birds, and kept constantly blowing on them, to drive them away. This was also an illusion of sensation.

3. A young lady became insane after the events of 1815. She had, or fancied a fixed pain in the crown of the head; and took a mortal hatred or dread of copper in every shape. She believed there was a worm in her head devouring her brain. M. Esquirol made a crucial incision on the scalp, and allowed the blood to flow. A piece of the fibrine was shewn her, and she was assured that this was the worm. An issue was established on the part. In three months she was cured of all her illusions. The same kind of insanity occurred in another individual, and was cured by the same means.

4. A general officer became insane after some domestic affliction. He had severe pains in his teeth, which he attributed to the sun. When severe, he raged at the great luminary, swearing he would exterminate Apollo and his chariot with his brave troops. Sometimes the pain attacked his knee, and then he fancied there was a thief there.

5. A lady became hypochondriacal, and hearing the temporal arteries beat, while lying on the pillow, she fancied that her brain was liquified, and running like torrents.

Gastric and intestinal pains, flatulency, and constipation produce great illusions in the minds of the insane. M. Esquirol opened a female, who had long affirmed that she had a living animal in her stomach. She had a cancer of that organ. A woman in the Salpêtrière believes that she has a whole regiment of soldiers in her belly. When the pain is violent, she roars out, and asserts that the soldiers are fighting, and wounding her with their bayonets.

We shall extract no more instances of illusions from internal sensations. We shall proceed to notice some arising from the external senses. Even in health the external senses are not infrequent sources of deception.

"The maniac hears a noise, he fancies some one speaks to him, and he answers as if questions had been addressed to him. If he hears several persons speaking, he thinks they are his friends, who are hastening to deliver him; or his subjects, who are come to raise him to the throne, and to proclaim him king.

The panaphobist, on the contrary, thinks that he is spoken to in a reproachful and menacing way; he takes an insignificant phrase for the sign of a plot raised against him; and he fancies he hears enemies, police agents, and murderers, concert together to arrest and to conduct him to prison or to the scaffold. If a door opens he imagines he is lost, and is about to become the prey of those who are seeking for him." 15.

After detailing several illustrations of illusion from the sense of hearing M. E. proceeds to those connected with the sense of sight.

"A lady, 23 years of age, afflicted with hysterical madness, used to remain constantly at the windows of her apartments during the summer. When she saw a beautiful cloud in the sky, she screamed out 'Garnerin, Garnerin, come and take me,' and repeated the same invitation until the cloud disappeared. She mistook the clouds for balloons sent up by Garnerin.

A cavalry officer imagined the clouds which he saw to be an army, led by Buonaparte, to make a descent upon England.

Insane persons often collect pebbles and fragments of glass, which they fancy precious stones, diamonds, or objects of natural history, and which they preserve with the greatest care." 18.

The sense of smell contributes to the illusions of the insane. Many persons smelling gas in the air, "fancy it unwholesome, and likely to poison them." There is something more than fancy for the first part of the illusion.

"Almost always at the commencement, and sometimes in the course, of mental diseases, the digestive functions are primarily or secondarily affected. Such patients perceive a bad taste in the food that is offered to them, which makes them conclude that it is poisoned, and they reject it with anger or with terror. This phenomenon gives rise to an aversion, on the part of the sick, to those persons who have the care of them, and which is still more marked towards those who are most dear and most devoted to them. What can be more dreadful than the fear of being poisoned by those we love?

These symptoms cease after a few days, either by diet or evacuations, when the gastric irritation is dispersed. The latter, which gives so much uneasiness to persons who are not in the habit of attending the insane, is by no means serious, and is very unlike the obstinate refusal of some monomaniacs, who will not eat, either to satisfy an absorbing idea, such as an expiation, the fear of neglecting some precept of honour or religion, or from a desire to terminate their existence.* The refusal to take nourishment, amongst the latter, should be combated by every possible means, in order to overcome a resolution which threatens their lives; whilst we should leave to themselves those patients who refuse their food because their taste and smell are perverted by the disordered state of the digestive organs." 25.

The sense of touch, so well calculated to correct the errors of other senses, sometimes deceives the insane. Several instances are related by our author, and some have been already adduced. The following are the conclusions to which M. Esquirol comes from the foregoing facts.

---

* "The difficulties which are experienced, in administering food to patients of this description, can hardly be conceived by those who have not had the management of them; by the use of the stomach-pump, however, these difficulties may, in some cases, be almost entirely obviated.

Some time since I had under my care an insane patient, about 30 years of age, who had been subject to epileptic fits from his boyhood. He had occasional attacks of violence, when he would remain for several days without taking any kind of nourishment. On one occasion, after abstaining longer than usual from food, it was thought advisable to give it to him against his inclination, and for that purpose I suggested the use of the stomach-pump, which I introduced with little difficulty. He was fed in this manner for two or three days, when, finding that resistance was in vain, he consented to take his food of his own accord.—*Translator*."

"1st. That illusions are caused by internal and external sensations.

2d. That they are the result of the sentient extremities, and of the re-action of the nervous centre.

3d. That they are as often caused by the excitement of the internal, as by that of the external senses.

4th. That they cannot be confounded with hallucinations, (visions,) since in the latter cases the brain only is excited.

5th. That illusions lead the judgment astray respecting the nature and cause of the impressions actually received, and urge the insane to acts dangerous to themselves and to others.

6th. That sex, education, profession, and habits, by modifying the reaction of the brain, modify also the character of the illusions.

7th. That illusions assume the character of the passions, and of the ideas which govern the insane.

8th. That reason dissipates the illusions of the man of sound mind, whilst it is not powerful enough to destroy those of the insane.

If by observation I have been able to elucidate a psychological phenomenon, but little appreciated, although common in delirium,—if the facts which I have related throw some light upon the still obscure history of the aberrations of the understanding, or if they furnish therapeutic views, applicable to the treatment of mental diseases, these observations will not be entirely without interest." 27.

### Medico-legal Question as to the Confinement of the Insane.

M. Esquirol is astonished that rules are not established for pointing out the cases which demand the suspension of liberty, in the persons of the insane. We should have thought Esquirol had been long enough in practice, and in the world, to know the difficulty of laying down precise rules upon such a point as this. We are astounded, however, at the annunciation that, in France, more than 15,000 individuals are deprived of their civil and political rights, as well as of their liberty, "without legal authority." Nevertheless, M. Esquirol agrees with all the most talented physicians of Europe, that confinement is the very best means of curing insanity.

"Amongst the numerous examples of insane persons, we meet with some individuals who recover their reason as soon as they leave their home, and lose it again on their return. When restored to their usual habits, and left to themselves, they give themselves up to excesses, experience contradictions, become angry at what they see, dread the duties and customs of the world, and the bustle of business; a thousand suspicions, troubles, and opposing pre-occupations and feelings, exalt or discourage them, and delirium breaks out. I have seen at the Salpétrière many women who could only be reasonable in the hospital, and who anxiously begged to be re-admitted, feeling, after passing some days in their family, that they were about to become ill again. Some of these patients, by returning soon enough, prevented the recurrence of the delirium; whilst others, leaving it until it was too late, were unable to escape the evil which they tried to avoid.

We have at Charenton a young man who has had many attacks of intermittent madness. Whilst he was out of the establishment these attacks were frequent; but he has now been there five years, and has not had one return of the disorder. For the last two years this patient has enjoyed all his reason; he is, however, kept within the house for fear of an attack, although, in other respects, he is quite at liberty." 36.

The reasons for confining the insane are thus summed up.

" 1. For their own security, for that of their families, and for the maintenance of public order.

2. To remove them from the influence of the external causes which have produced their disorder, and may be likely to protract it.

3. To overcome their resistance to curative means.

4. To submit them to a regimen appropriate to their situation. And,

5. To make them resume their moral and intellectual habits." 71.

There are objections, however, to the rule. If the patient be furiously mad, every body sees the necessity of confinement.

" But shall we confine the insane patient, who enjoys a great portion of his reason, who has only partial delirium, and who retains almost all his moral sensibility ? Will not the opposition which he is about to experience deprive him of that portion of intelligence which remains ? Is it not cruel to deprive a patient of the attentions of his family, and to separate a miserable being, who is loaded with grief, from the objects of his affections ? Shall we remove the panaphobist from the relations and friends whom he regards as his natural protectors, and deprive him of his liberty who is afraid of the police, prisons, chains, &c. &c. ? How many more objections may not be made ? Experience has answered and has proved, that the insane rarely get well in the midst of their own families, and that their cure is more rapid and more certain when they are treated away from home. We dread the contact of their companions in misfortune, lest, by imitation, the ideas and actions of those already in confinement should augment their delirium. We are afraid that patients in this state may experience the same shock which is felt by other persons, forgetting that their sensibility is perverted, and that they do not feel like persons enjoying the plenitude of health. But who will dare to assert that confinement has never been prejudicial ? I frankly own that it sometimes is so ; for it partakes of the nature of those things, the best of which are not always free from inconveniences. What must we then conclude ! That confinement should not be abused, that we should not apply it too generally, nor too exclusively ; and that it should be prescribed only by the experienced physician.

Every patient who is delirious ought not to be confined; for acute and febrile delirium often puts on the appearance of insanity. It is easy to be imposed upon in this respect; and the error is not a trifling one, for it compromises the health of the patient, and exposes the medical attendant to censure. When we are called to a patient who is delirious, we ought not to be in a hurry to give an opinion. I have attended some cases in which I have objected to confinement, although it appeared highly necessary, on account of the violence of the delirium. This precaution would be superfluous at the commencement of a second attack of madness, or of intermittent insanity, and it would be prejudicial when there is a tendency to suicide.

It does not follow that confinement should be prescribed for all insane persons ; for if the delirium is partial or transitory ; if it relates only to objects of indifference, and is unaccompanied with violent passion ; if the patient has no aversion to his home, nor to the persons with whom he lives, and his delirium is independent of his domestic habits ; if his real or imaginary causes of excitement are not to be found in the bosom of his family ; if the fortune or life of the patient, or of his friends, are not compromised, and he submits to the proper means of cure; in all these cases confinement may be useful, but is not indispensable. If the patient, retaining a large proportion of his intellect, has a great attachment to his relations, it is to be feared that confinement might aggravate the disease.

Confinement is indispensable in mania, and also in monomania, when the patients are actuated by pride, love, or jealousy. Lypemaniacs, who are full of imaginary terrors, such as panaphobists, and patients with a tendency to suicide, should also be confined. The latter are cunning, and crafty, and know how to defeat the most active superintendence. Confinement alone can insure the preservation of their lives; indeed it is always necessary to be on the watch for their safety.

Persons in a state of fatuity have only need of attention, and may remain with their friends unless peculiar circumstances, involving other parties, should render separation necessary; a pregnant woman, who is easily excited, would run some risk, perhaps, in living constantly with a person in a state of fatuity, although he might be very quiet. The presence of an insane person, in a family composed of several children, especially young ladies, might become a predisposing cause of mental diseases, and confinement would be therefore necessary.

Idiots have nothing to hope from confinement: if they are shut up it is only to preserve them from the accidents to which their condition exposes them; to remove them from the raillery of the ignorant, and to prevent their becoming the instruments which malefactors have sometimes made use of for criminal purposes.

The insane poor ought generally to be confined, as their relations are without the means of procuring proper attendance for them.

Whenever an insane patient, whatever may be the character of the disease, has been treated at home for a longer or a shorter period, his health requires that confinement should be tried, as one of the most powerful means of cure." 75.

With this extract, we conclude our notice of the work. With the exception of a few literal or verbal errors, the translation is a very fair one.

